# COVID-19 and surgical education: Every cloud has a silver lining

**DOI:** 10.1016/j.amsu.2020.08.017

**Published:** 2020-08-25

**Authors:** Michail Sideris, John Gerrard Hanrahan, Vassilios Papalois

**Affiliations:** a Women's Health Research Unit, Queen Mary University of London, London, UK; b Addenbrookes Hospital, Cambridge, UK; c Imperial College London, London, UK

**Keywords:** Surgical education, Innovation in education, COVID19

## 1 Existing landscape

Much of today's education and training is purportedly provided “on the job”, where trainees are expected to accumulate skills and experience whilst providing patient-centred care. Yet, the priority of clinical practice is patient safety and care, taking precedent over educational needs of trainees, often resulting in administrative tasks of such care eroding educational opportunities. Although surgeons' first and foremost duty is to look after patients, they are also obliged to entitle time for education, training, research and management. This could be even accommodated in the ward round or theatre settings as examples - but does this happen these days?

Additionally, medical education and training are currently completely disengaged from workforce planning. We design curricula and training programmes without knowing what are the real current and, even more so, future needs in our profession. You ask surgical trainees what they would like to dedicate their careers to, and they come up with socialites and competencies that will be soon non existing. To-date, nobody has clearly addressed the importance of appraising first what kind of surgeons (or medics altogether) we will need during the next 2-3 decades, and tailoring education and training accordingly.

## 2 The effect of COVID-19 pandemic

Since December 2019, the COVID-19 pandemic has rapidly changed healthcare priorities and delivery. In our recent systematic review [1], we described how most educational activities were ceased in order to reiterate our forces to tackle the pandemic. However, it became necessary for existing alternative technologies and approaches to provide other means of delivering education. Examples included tele-conferences, webinars, social media and simulation which allowed quick action and minimise the disruption to trainee education. This was a first response which clearly provided an impetus for pedagogical novelties, which could be a stepping-stone for a legacy in medical education.

## 3 Examples from previous disasters in our history

The world has been through jarring recessions in the past. In 1947, shortly after the end of World War II, Bell Labs invented the point-contact transistor, and since then technology has driven a massive economic expansion which was set to continue and change the world [2]. Equally, Artificial Intelligence and its application in medicine are set to drive a rapid evolution – an evolution potentially accelerated by COVID-19 to meet new educational demands.

## 4 Taking things forward

Ancient Greeks believed that any ill wind has to blow something good. COVID-19 has been a seismic change in medical and surgical education, and perhaps the “point zero” to consider shifting changes for the future. Despite the short-term detrimental consequences, natural disasters like a pandemic have the power to make medical community re-evaluate the delivery and flexibility of training curricula and programmes, and allow trainees to genuinely learn via dealing with those massive challenges.

The 20th century concept of the successful surgeon (specialist overall) as one who super-specialises in one domain and also conducts sophisticated research in that domain that results in great publications, is gradually fading. What we will need are clinical leaders with expertise but also flexible clinicians who hold a basic generic skill set which is always in high demand. This generic skillset can be a useful basis for clinicians to adapt in different circumstances and changing realities, to meet the changing health system demands. This has been exemplified by the COVID-19 pandemic where a huge cohort of the working force have skills which are irrelevant to the basic skills needed to fight this catastrophe.

In conclusion, on direct questioning as to whether COVID19 is detrimental for medical education, the short-term answer is yes. However, long term it can act as the missing dynamite to change the current “tired” and overwhelmed landscape and reconsider a novel approach for medical and surgical curricula.

## 5 Place of study

Imperial College London, Directorate of Renal and Transplant Services, 4th Floor Hammersmith House, Hammersmith Hospital, Du Cane Road, London W12 OHS, United Kingdom.

## Ethical approval

None required.

## Funding

There are no funders to report for this submission.

## Informed consent

No need for informed consent, as this study did not involve individual participants.

## Consent for publication

For this type of study consent for publication is not required.

## Provenance and peer review

Not commissioned, editor reviewed.

## CRediT authorship contribution statement

**Michail Sideris:** Writing - original draft. **John Gerrard Hanrahan:** Writing - original draft. **Vassilios Papalois:** Writing - original draft.

## Declaration of competing interest

The authors declare that they have no conflict of interest.
